# Diversity patterns of soil microbial communities in the *Sophora flavescens* rhizosphere in response to continuous monocropping

**DOI:** 10.1186/s12866-020-01956-8

**Published:** 2020-08-31

**Authors:** Haiying Lei, Ake Liu, Qinwen Hou, Qingsong Zhao, Jia Guo, Zhijun Wang

**Affiliations:** 1 Faculty of Biology Sciences and Technology, Changzhi University, Changzhi, Shanxi 046011 P. R. China; 2 Department of Chemistry, Changzhi University, Changzhi, Shanxi 046011 P. R. China

**Keywords:** *Sophora flavescens*, Continuous monocropping, Rhizosphere soil, Microbial community, Soil physicochemical property

## Abstract

**Background:**

Continuous monocropping can affect the physicochemical and biological characteristics of cultivated soil. *Sophora flavescens* is a valuable herbal medicine and sensitive to continuous monocropping. Currently, diversity patterns of soil microbial communities in soil continuous monocropping with *S. flavescens* have not been extensively elucidated.

**Results:**

In this study, comparative 16S rDNA and internal transcribed spacer (ITS) MiSeq sequencing analyses were used to examine the taxonomic community structure and microbial diversity in nonrhizosphere soil (CK) and rhizosphere soils (SCC, TCC, and FCC) sampled from fields that had undergone two, three, and five years of continuous monocropping, respectively. Among the microbial communities, a decreased abundance of Acidobacteria and increased abundances of Proteobacteria and Bacteroidetes were found with the increase in monocropping years of *S. flavescens*. As the continuous monocropping time increased, the diversity of the bacterial community decreased, but that of fungi increased. Redundancy analysis also showed that among the properties of the rhizosphere soil, the available phosphorus, organic matter, total nitrogen, and sucrase had the greatest impacts on the diversity of the rhizosphere microbial community. Moreover, a biomarker for *S. flavescens* soil was also identified using the most differentially abundant bacteria and fungi in soil samples.

**Conclusions:**

Our study indicates that long-term monocropping exerted great impacts on microbial community distributions and soil physicochemical properties. The relationship between microbial community and physicochemical properties of rhizosphere soil would help clarify the side effects of continuous *S. flavescens* monocropping. Our study may aid in uncovering the theoretical basis underlying obstacles to continuous monocropping and provide better guidance for crop production.

## Background

The rhizosphere is a special microecosystem formed by interactions among plants, soil, and microorganisms [1, 2]. Rhizosphere microorganisms participate in a series of processes, such as the decomposition of soil organic matter, formation of humus, and transformation and circulation of soil nutrients [3]. There is an adaptive coevolutionary relationship between rhizosphere soil microorganisms and root systems that also builds the reciprocal relationship between plant and soil microorganisms [4, 5]. Rhizosphere population structures and activity changes are important indexes for measuring soil quality and maintaining soil fertility and crop yields [3]. Plants can also affect the nutrient content of the rhizosphere and other soil physicochemical properties through root activity, which leads to significant differences in the composition and diversity of the rhizosphere and nonrhizosphere soil microbial communities [6].

Agricultural managements, including continuous monocropping, can affect physicochemical and biological characteristics of cultivated soil [7]. Many studies have shown that in comparison to intercropping, continuous monocropping has obvious effects on organic matter, soil enzyme activities, microbial communities, and crop productivity [8–10]. As a major determinant, plants can shift their soil microbial communities through the accumulation of litter and rhizodepositions in the soil under continuous monocropping conditions, thereby altering subsequent plant growth [11, 12]. Moreover, other factors such as phytotoxic compounds, soil-borne pathogens, soil physicochemical property changes, and nutrient deficiency can also contribute to the diversity of microbial communities [7, 13, 14]. Many plants have been reported to suffer from high mortality, declines in yield and quality, and stunting caused by continuous monocropping systems, such as notoginseng [15], soybean [16, 17], rice [18], peanut [7], and cucumber [19]. For these reasons, agricultural activities should be paid more attention, especially when exploring the underlying mechanism of continuous monocropping and soil sickness.

*Sophora flavescens* is a traditional Chinese medicine from Fabaceae. It is widely used for the treatment of various diseases, such as viral hepatitis, cancers, viral myocarditis, gastrointestinal hemorrhage, and skin diseases [20]. The active components of this species include matrine and oxymatrine, which have good anticancer activities [21–23]. Generally, medicinal plants are continuously monocropped in a certain region due to limited arable land, economic benefits, and regional agro-industrialization [24, 25]. However, in practice, different degrees of soil sickness exist for *S. flavescens* soil fertility, which seriously affects the yield and quality of medicinal materials. During continuous monocropping, plant roots repeatedly release the same types of exudates for many years. This phenomenon occasionally leads to significant colonization and infection by certain beneficial or pathogenic microorganisms, which can utilize these substrates [26]. Thus, it is important to detect beneficial microorganisms in the rhizosphere of *S. flavescens* to improve its soil fertility. Moreover, overuse of chemical fertilizers to control product losses also creates severe problems and may deteriorate the quality of medicinal plant products. Therefore, it is necessary to understand microbial ecological patterns and their relationships with environmental factors during continuous monocropping of *S. flavescens*.

In this study, we used the combined sequencing of 16S rDNA and fungal internal transcribed spacer (ITS) MiSeq to highlight the changes in microbial diversity and community structure of *S. flavescens* rhizosphere soil in response to continuous monocropping system (2, 3, and 5 years of continuous monocropping). The aims of this study were to 1) evaluate the effects of continuous monocropping on soil quality, 2) investigate diversity patterns of soil bacterial and fungal communities in the *S. flavescens* rhizosphere in response to continuous monocropping, and 3) study the mechanisms underlying continuous monocropping obstacles by identifying relationships between taxonomic patterns and soil physicochemical properties. This study will help to elucidate the relationships among *S. flavescens*, soil, and rhizosphere microorganisms and provide a theoretical basis for revealing the mechanism underlying continuous monocropping obstacles and better guidance for monocropping.

## Results

### Soil physicochemical properties were affected by continuous monocropping of *S. flavescens*

The results of a comparative analysis of soil physicochemical properties among the four sampling sites, SCC, TCC, FCC (second-, third- and fifth-year continuous monocropping, respectively), and CK (control) sites are shown in Table 1. All the soils were alkaline (pH value, 7.58 ~ 8.20), and the CK soil had the highest pH value, which was significantly different from the SCC and TCC rhizosphere soil. The available phosphorus (AP) content was lowest in CK soil and highest in SCC rhizosphere soil. The contents of soil organic matter (OM) and total nitrogen (TN) were lowest in SCC soil and highest in TCC soil (TCC > FCC > SCC). The sucrase content was lowest in FCC soil and highest in CK soil. The urease content was lowest in TCC soil and highest in FCC soil. Among these parameters, the OM and sucrase contents did not significantly differ for different years of continuous cropping with *S. flavescens*, and the other four indexes showed significant differences in rhizosphere soil for different years of continuous monocropping (Table 1).

**Table 1:** The rhizosphere and bulk soil physical and chemical properties of *S. flavescens*

sample	pH	AP (mg/kg)	OM (mg/kg)	TN (g/kg)	Sucrase (mg/g)	Urease (mg/g)
SCC	7.59 ± 0.20b	12.27 ± 0.19a	115.63 ± 10.52b	0.03 ± 0.01b	1.16 ± 0.17a	0.43 ± 0.03b
TCC	7.58 ± 0.04b	8.32 ± 0.85b	141.27 ± 10.02a	0.63 ± 0.11a	1.37 ± 0.14a	0.38 ± 0.07ab
FCC	8.13 ± 0.14a	2.19 ± 0.34c	127.22 ± 5.41a	0.07 ± 0.01b	1.12 ± 0.09a	0.67 ± 0.05a
CK	8.20 ± 0.01a	1.57 ± 0.48c	136.35 ± 14.04a	0.10 ± 0.05b	1.42 ± 0.09a	0.63 ± 0.07ab

Different lowercase letters indicate significant differences between different samples (*P* < 0.05)

### Field microbial community structure variations with continuous monocropping time

**Fig. 1:**
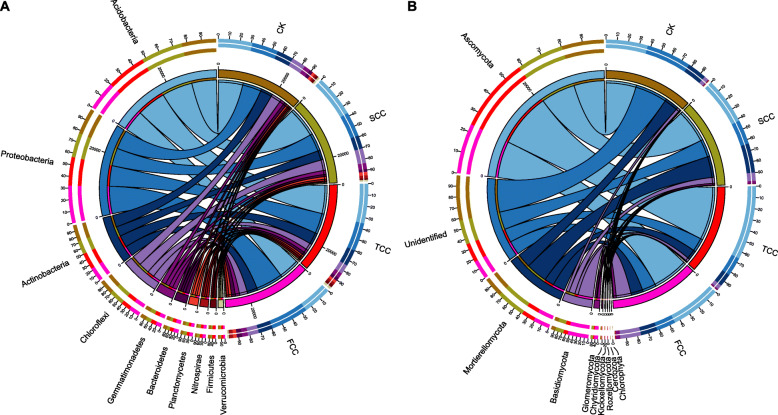
Distribution of the ten most abundant bacterial (**a**) and fungal (**b**) phyla of rhizosphere soil after various years of continuous monocropping with *S. flavescens*. The bar length on the outer ring represents the percentage of each phylum in each sample

For the bacterial communities, the operational taxonomic units (OTUs) from four soil sites were found to belong to 40 phyla, 101 classes, 129 orders, 233 families, and 318 genera. The composition of the bacterial community at the phylum level and its phylum abundances are shown in Fig. 1a and S1A. The top 10 relatively abundant bacterial phyla over all samples included Acidobacteria, Proteobacteria, Actinobacteria, Chloroflexi, Gemmatimonadetes, Bacteroidetes, Planctomycetes, Nitrospirae, Firmicutes, and Verrucomicrobia. The sum of these phyla accounted for more than 93% of the bacteriome. Acidobacteria and Proteobacteria accounted for the largest proportion (more than 51%). The abundance of Acidobacteria in the communities of SCC, TCC, and FCC soils presented a decreasing trend with increasing time (Fig. S1A, 34.9–24.3%). The abundance of Proteobacteria increased in SCC soil (21.0%) compared with CK soil and reached its highest level in the FCC community (31.9%). The abundance of Actinobacteria was lower in the SCC community than other samples but with only small differences. Finally, the abundance of Bacteroidetes in the community presented an increasing trend from SCC to FCC soil (Fig. S1A, 2.7–4.6%).

For the fungal communities, OTUs detected from the four soil samples belonged to 13 phyla, 35 classes, 89 orders, 170 families, and 280 genera. Nine phyla and one unidentified phylum were identified from the soil samples (Fig. 1b, S1B), which accounted for over 99% of the fungal sequences. The nine determined phyla were Ascomycota, Mortierellomycota, Basidiomycota, Glomeromycota, Chytridiomycota, Kickxellomycota, Rozellomycota, Cercozoa, and Chlorophyta. The fungal community of CK soil diverged from those of rhizosphere soils in abundances of both Ascomycota and unidentified phylum; the former dominated in rhizosphere soil, especially in TCC soil (70.0%), and the latter dominated in CK soil (39.6%). The abundance of Basidiomycota was highest in the FCC community (18.5%). Chlorophyta was present only in CK soil with a low abundance (0.1%), but it was not detected in other rhizosphere soils. Moreover, the abundance of Rozellomycota in the community increased from SCC to FCC soil (Fig. S1B, 0.04–0.34%).

**Fig. 2:**
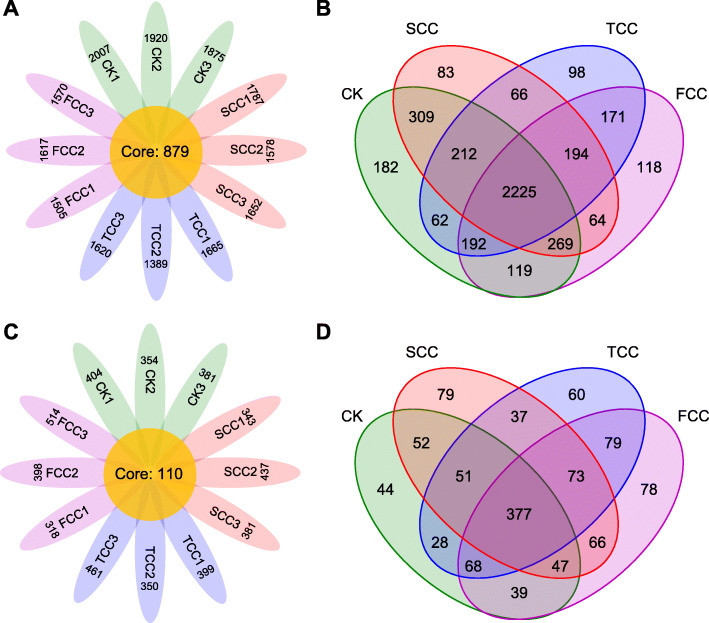
OTUs of microbial communities in rhizosphere and CK soils. **a** Flower plots showing the number of sample-specific OTUs (in the petals) and core OTUs (in the center) for all samples. **b** Venn diagram of OTUs observed as unique or shared among the four sites. The number of each site contains all the identified OTUs from all replicates. The above description also applies to **c** and **d**

Accordingly, the OTUs identified in all analyzed samples were regarded as the core OTUs, and those identified in at least one sample were defined as pan-OTUs [27]. Here, core and pan-OTUs were identified for all soil samples (Fig. 2). In total, 879 core OTUs (Fig. 2a, Table S1) and 4364 pan-OTUs were identified in the bacterial community (Fig. 2b), and 110 core OTUs (Fig. 2c, Table S1) and 1178 pan-OTUs were identified in the fungal community at all sites (Fig. 2d). There were 2225 bacterial OTUs shared by all four samples and 2419 OTUs shared by SCC, TCC, and FCC soils. The number of unique bacterial OTUs for these four sites was as follows: 182, 83, 98, and 118 for CK, SCC, TCC, and FCC soils, respectively (Fig. 2b). A total of 377 fungal OTUs were identified as common OTUs for all samples (Fig. 2d). Four hundred and fifty fungal OTUs were shared by SCC, TCC, and FCC soils. There were 44, 79, 60, and 78 fungal OTUs unique to CK, SCC, TCC, and FCC soils, respectively. Before and after continuous monocropping, the bacterial OTU numbers for CK, SCC, TCC, and FCC soils were 3570, 3422, 3220, and 3350, respectively (Fig. 2b), and the fungal OTU numbers were 706, 782, 773, and 827, respectively (Fig. 2d).

### Microbial diversity was influenced by the continuous monocropping time

**Fig. 3:**
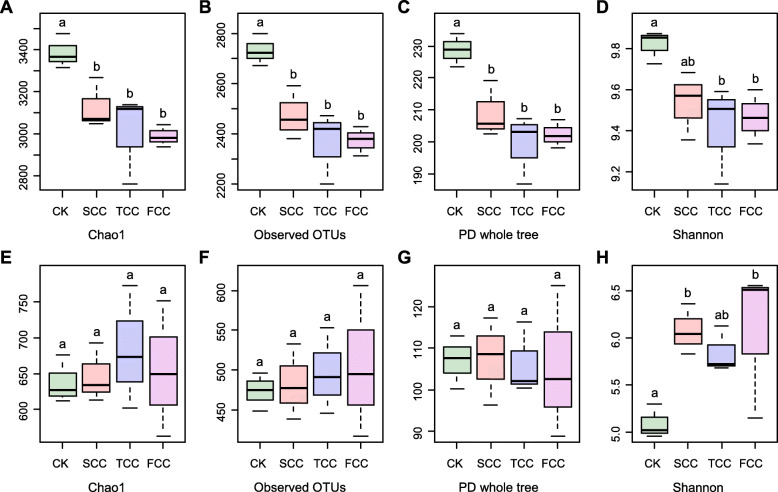
Alpha diversity of soil microbial communities at sites after various years of continuous monocropping with *S. flavescens*. The top and bottom panels show the estimates of bacterial and fungal alpha diversity of each sample, respectively. Different lowercase letters above the boxplots indicate significant differences between different samples according to one-way ANOVA with Duncan’s multiple range test (*P* < 0.05)

The alpha diversity represents the measurement of within-community microbial diversity, which can be used to compare the diversities of *S. flavescens* rhizosphere soil among sites during different continuous monocropping times (Fig. 3). For the bacterial community, the Chao1 index values of CK, SCC, TCC, and FCC rhizosphere soils were 3384.07, 3128.26, 3006.66, and 2988.44, respectively (Fig. 3a-d). According to a Shannon index analysis, the bacterial species richness was highest (9.82) for the rhizosphere soil of CK, followed by SCC (9.54), TCC (9.41), and FCC (9.47) soils (Fig. 3d). The diversity index results showed that the highest bacterial diversity of *S. flavescens* rhizosphere was found in CK soil and was lowest in TCC soil. For fungi, the trends in rhizosphere richness and diversity index were basically opposite for the three continuous monocropping with *S. flavescens* periods (Fig. 3e-h). The Chao1 diversity and Shannon index values of FCC rhizosphere fungi were 654.75 and 6.07, respectively, followed by those of SCC rhizosphere fungi, which were 646.66 and 6.08, respectively, while the CK soil showed the lowest values of 637.93 and 5.09 (Fig. 3e, h). The results showed that the number of years of continuous monocropping of *S. flavescens* and the presence of *S. flavescens* itself were the main factors affecting the diversity of rhizosphere fungi.

**Fig. 4:**
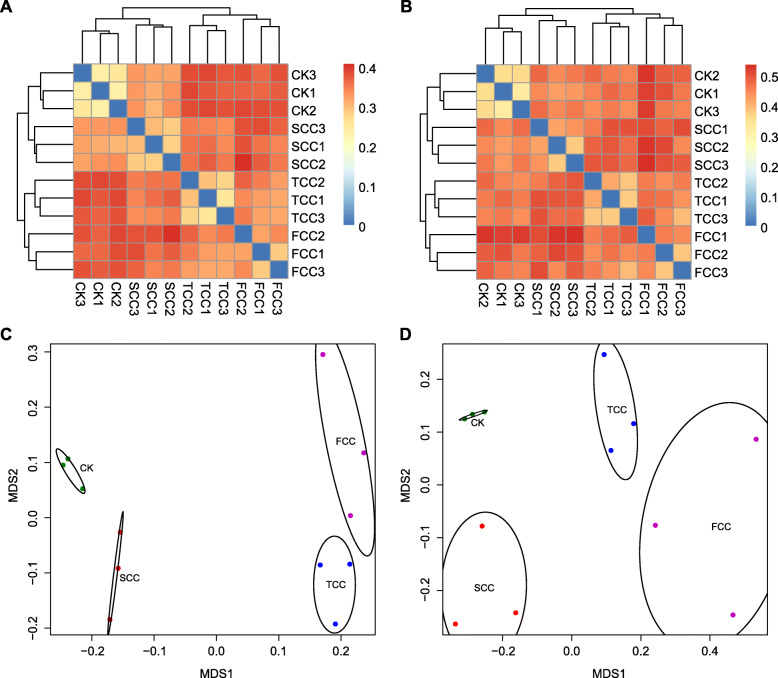
Unweighted UniFrac clustering and NMDS of microbial communities in *S. flavescens* rhizosphere and CK soils. A and B show the unweighted UniFrac heatmaps for all samples, demonstrating the similarity of the microbial community structure between rhizosphere and CK soils. C and D show two-dimensional, nonmetric multidimensional scaling (NMDS) results representing the bacterial and fungal communities present in the four *S. flavescens* sampling sites

To better display the distance relationship between multiple samples, the microbial β diversity was further assessed based on the unweighted UniFrac distance matrix [27]. The biological replicates clustered together, samples from the CK soil clustered with those from SCC soil, and samples from the other two sites (TCC and FCC) clustered together (Fig. 4a, Fig. S2). Importantly, great divergences in β diversity were identified between short-cropped and long-cropped sites (Fig. 4a). For the fungal community, a similar pattern was observed (Fig. 4b, Fig. S2). Both the bacterial and fungal community compositions varied among different samples, which was also graphically illustrated in the nonmetric multidimensional scaling (NMDS) [28] ordination and principal components analysis (PCA) (Fig. 4c, d and S2). Moreover, the community composition was apparently affected by the continuous monocropping time and marginally influenced by the sampling sites.

### Correlating physicochemical properties with microbial diversity

**Fig. 5:**
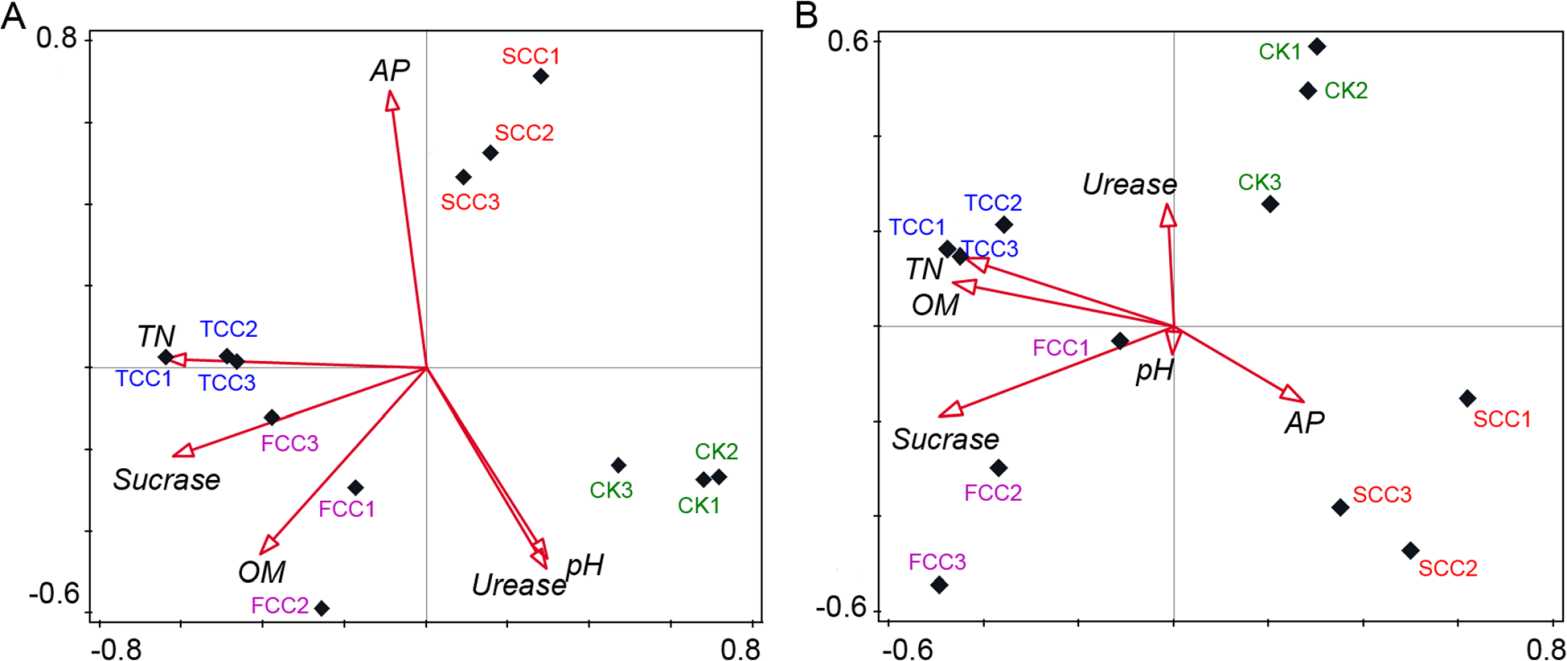
RDA plots of the bacterial (**a**) and fungal (**b**) communities with respect to environmental variables in the root zone of *S. flavescens*. AP (mg/kg), OM (g/kg), sucrase (mg/g), TN (g/kg), urease (mg/g)

Soil physicochemical properties (the pH and AP, OM, TN, sucrase, and urease contents) were significant explanatory factors determining the observed clustering pattern of soil microbial communities for different years of continuous monocropping (Fig. 5a). To better understand the clustering and separation of samples caused by environmental factors, redundancy analysis (RDA) was conducted for both soil bacterial and fungal communities (Fig. 5). The bacterial community in the rhizosphere of SCC soil was related to the AP, that of TCC soil was related to soil TN, and that of FCC soil was related to sucrase and OM. The soil pH and urease determined the pattern of the CK microbial communities (Fig. 5a). As expected, these six soil indexes also contributed to the composition of the fungal community (Fig. 5b). AP was identified as a primary explanatory factor responsible for the observed clustering pattern in the SCC rhizosphere fungal community. The TCC rhizosphere fungal community was strongly related to soil TN, urease, and OM. The FCC rhizosphere fungal community was related to soil sucrase and pH. However, the driving factor for the formation of the CK fungal community was not identified. These results showed that for bacteria and fungi, microbial diversity in SCC soil was positively correlated with AP, and the diversities of the TCC and FCC rhizosphere communities were positively correlated with TN, OM, and sucrase. Moreover, the continuous monocropping time showed a lower correlation with soil pH and urease in fungal communities than in bacterial communities. AP is closely related to SCC soil and served as a main explanatory factor for the diversity of rhizosphere bacterial and fungal communities in SCC soil.

### Discovery of biomarkers in microbial communities over different continuous monocropping times

**Fig. 6:**
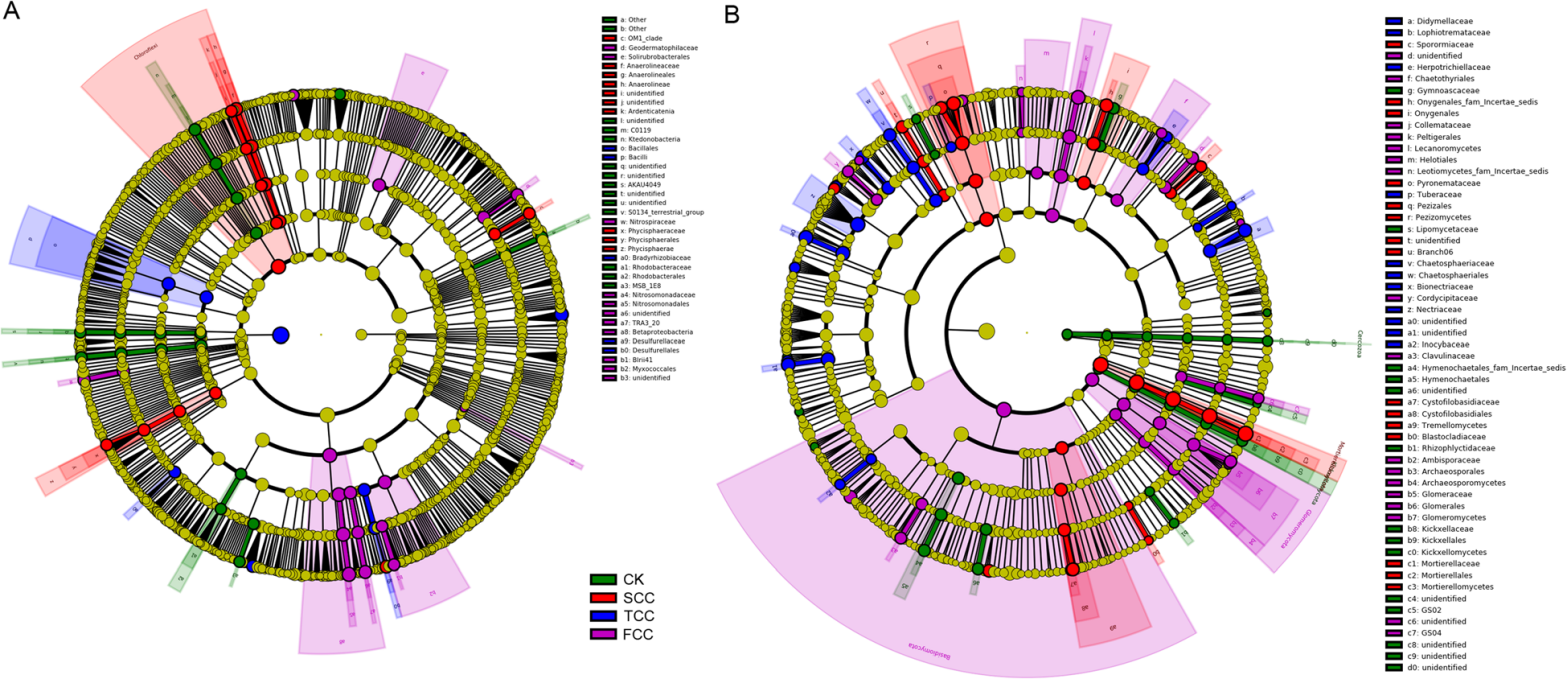
LEfSe analysis of bacterial 16S rDNA (**a**) and fungal ITS rDNA (**b**) sequences with different abundances between soil samples. The circles radiating from inside to outside represent the taxonomic level from phylum to genus. Each small circle at a different classification level represents a classification at that level, and the diameter of the small circle is proportional to the relative abundance of that taxon. The nonsignificantly different species are uniformly colored yellow, and the different species biomarkers are colored the same as the groups. Green nodes represent the microbial groups that play an important role in the control group, and red, blue, and purple nodes represent the microbial groups that play an important role in the SCC, TCC, and FCC groups, respectively. The names of species represented by letters are shown in the legend on the right

For the bacterial community, a linear discriminate analysis (LDA) effect size (LEfSe) [29] analysis identified 21, 16, 9, and 17 biomarkers for the CK, SCC, TCC, and FCC fields, respectively (Figs. 6a, S3A). The most abundant bacteria from CK soil belonged to Rhodobacterales. SCC fields were abundant in the bacteria Chloroflexi and Phycisphaerales. Bacilli were significantly enriched in TCC soil. Biomarkers in samples from the FCC field mainly comprised members of the Betaproteobacteria, Myxococcales, and Solirubrobacterales (Fig. 6a). For the fungal community, the LEfSe analysis identified 29, 27, 23, and 32 biomarkers for the CK, SCC, TCC, and FCC fields, respectively (Figs. 6b, S3B). For the CK sample, fungi that were relatively abundant included members of the Kickxellomycota and Hymenochaetales. Biomarkers in samples from the SCC field included members of the Tremellomycetes and Pezizomycetes. For the TCC field, fungi that were differentially abundant included members of the Nectriaceae, Didymellaceae, and Herpotrichiellaceae. The most differentially abundant fungi from the FCC field mainly included members of the Basidiomycota and Glomeromycota (Fig. 6b).

## Discussion

Continuous monocropping results in dynamic transformation of soil microorganisms [17, 30]. Because at least 2 years of *S. flavescens* cultivation is required to meet the criterion for effective ingredients used in clinical therapeutics [31], our study excluded the soil samples from the first year of *S. flavescens* monocropping. As shown in Fig. 1, the common phyla for both bacteria and fungi were similar across rhizosphere soils, but their relative abundances were quite different. Among them, a decreased abundance of Acidobacteria and increased abundances of Proteobacteria and Bacteroidetes were found with the increase in monocropping years of *S. flavescens*. Similar to the results of previous studies, continuous monocropping can easily reduce the abundance of bacteria and increase the abundance of fungi“ or “number of bacterial species and increase the number of fungal species (trend of a transformation from “bacterial soil” to “fungal soil”) [17, 30], which can lead to soil failure, disease, and pest problems [32]. Accordingly, the majority of the soil fungi were phytopathogenic microorganisms and not conducive to plant growth [33], indicating that increasing the continuous monocropping years decreases soil fertility and increases plant disease incidence [34]. These fungi may be actively filtered by plant roots, resulting in a higher abundance of fungi in rhizosphere than nonrhizosphere soil [33]. The diversity analysis of the bacterial community composition of *S. flavescens* rhizosphere soil also showed obviously distinct microbial communities in rhizosphere and CK soil (Figs. 3, 4) and very similar rhizosphere soil communities.

Continuous monocropping can also lead to soil sickness, nutrient imbalances, and even yield reduction [17, 35]. Soil physicochemical properties are important factors for soil fertility and can affect the population structure and function of rhizosphere microorganisms [36, 37]. Soil pH can affect the activities of soil rhizosphere microorganisms and the release, transformation, and migration of soil nutrients [3, 17]. However, soil pH plays a less important role in the variance in fungal community structure (Fig. 5b) due to the broader suitable pH range for fungal growth than for bacteria [38]. The RDA results showed that the community structures of rhizosphere bacteria and fungi were positively correlated with soil AP, TN, sucrase, and OM, while the community structure of CK bacteria and fungi was positively correlated with soil urease and pH (Fig. 5). Soil enzymes are another important factor for soil metabolism, performing roles in nutrient conversion, energy metabolism, and pollutant detoxification [39, 40]. Sucrase hydrolyzes sucrose and can reflect soil organic carbon conversion ability, while urease hydrolyzes urea and can affect soil nitrogen metabolism [41]. This study showed a positive relationship between the diversity and composition of bacteria to pH and urease, which is also consistent with the results of previous studies [42]. The level of soil phosphorus content to a certain extent reflects the storage and supply capacity of phosphorus in soil [43]. The differences between rhizosphere and CK communities may also be associated with the higher content of AP in rhizosphere soil than in CK soil [17]. The pH value and TN and sucrase contents of rhizosphere soil were lower than those of CK soil, which led to an increase in abundance of some specific groups.

The diversity of bacteria was lower but the diversity of fungi was higher in rhizosphere than CK soil (Fig. 3), in agreement with results of a previous study [44]. The base soil of the medicinal planting experiment revealed a lack or even an extreme lack of TN (Table 1); this lack was conducive to bacterial reproduction and thus increased bacterial richness and diversity. Our study implies that to ensure soil nutrient abundance in plantation areas, the amount of organic fertilizer must be increased, nitrogen and phosphorus levels scientifically balanced, appropriately increasing the amount of nitrogen fertilizers, and controlling the amount of phosphate fertilizers are required. Plant rhizosphere bacterial diversity is closely related to plant growth and development [45].

In summary, we studied the diversity patterns in the microbial community abundance and composition in rhizosphere soil of continuously monocropped *S. flavescens*. With increasing continuous monocropping time, the diversity of the bacterial community decreased but that of fungi increased, indicating that long-term monocropping could significantly alter the microbial community structures. The correlation between the microbial community and physicochemical properties of rhizosphere soil can be used to clarify the relationship between continuous monocropping with *S. flavescens* and soil degradation and provide a theoretical basis for *S. flavescens* cultivation and standardized planting. Understanding the diversity patterns of microbial communities for different monocropping and management systems will help clarify the relationship between continuous planting of *S. flavescens* and soil degradation [46, 47].

## Conclusion

This is the first report to highlight the influence of a continuous monocropping system on soil microbial diversity and community structure in *S. flavescens* plantations. The results showed that in *S. flavescens* rhizosphere soil, the dominant bacterial phyla were Acidobacteria and Proteobacteria and the dominant fungal phyla were Ascomycota and Mortierellomycota. As the continuous monocropping time increased, the diversity of the bacterial community decreased but that of fungi increased, indicating that long-term monocropping exerted great impacts on microbial community distributions. RDA further demonstrated the primary functions of soil TN, sucrase, and OM in shaping the distributions of bacterial and fungal communities over 5 years of continuous monocropping. Moreover, a biomarker of *S. flavescens* soil was also identified to determine the most differentially abundant bacteria and fungi in soil samples. This study provides insight into the mechanism underlying obstacles in continuous monocropping systems and will be helpful for improving the yield and quality of medicinal plants.

## Methods

### Sample collection

*S. flavescens* rhizosphere samples were collected from a Standardized Planting Base of Sophorae in Zhendong Chinese Herbal Development Company (longitude/latitude: E113°01′8.50″/N36°01′30.71″; Elevation: 944 m) using conventional methods in July 2018. The soil samples of SCC, TCC, and FCC sites were collected from the rhizosphere areas of *S. flavescens* after the second, third, and fifth year of continuous monocropping, respectively. The soil samples of the CK site were collected from nonplanting areas around the *S. flavescens* planting areas. Each sample was collected from five individual plants within a field of 100 m^2^. These five individuals were approximately 5–10 m from one another. Large clumps of soil not adhering to the roots were removed, and only the soil closely attached to the root surface was collected as a rhizosphere sample. Each site, with 3 replications, was further divided into two parts: one part was frozen immediately in liquid nitrogen and stored at − 80 °C until DNA extraction, while the other part was dried naturally in a room and then analyzed for physical and chemical indexes.

### Determination of soil physical and chemical indexes

After air-drying the samples and passing them through a 2-mm sieve, the pH value, AP, OM, TN, sucrase, and urease contents of the collected soils were measured accordingly. Specifically, the soil pH value was determined using a pH meter (Mettler Toledo, USA) in a soil water suspension (1:5 w/v) after shaking for 30 min [48]; AP was determined according to the method reported by Ryan et al. [49]; OM was determined by the potassium dichromate method [50]; TN was determined by the Kjeldahl method [51]; sucrase content was determined by 3,5-dinitrosalicylic acid colorimetry [52]; and urease content was determined by the indophenol blue colorimetric method [52].

### DNA extraction, PCR amplification, and sequencing

A PowerSoil DNA Isolation Kit (MoBio, USA) was used to extract genomic DNA from rhizosphere soil samples following the manufacturer’s instructions. DNA purity was quantified by a NanoDrop spectrophotometer and checked by 0.8% agarose gel electrophoresis. The V3-V4 hypervariable region of the bacterial 16S rDNA was amplified with primers 341F and 806R (F5’-ACTCCTACGGGAGGCAGCAG-3′, R5’-GGACTACHVGGGTWTCTAAT-3′) [53, 54]. For each soil sample, a 10-digit barcode sequence was added to the 5′ end of the forward and reverse primers (provided by Auwigene Company, China). PCR was conducted on a Mastercycler Gradient Thermocycler (Eppendorf, Germany) using 50-μL reaction volumes containing 5 μL 10× Ex Taq Buffer (Mg^2+^ plus), 4 μL 12.5 mM dNTP (each) mix, 1.25 U Ex Taq DNA polymerase, 2 μL template DNA, 200 nM barcoded primers 967F and 1406R (each), and 36.75 μL ddH_2_O. The cycling parameters were 94 °C for 2 min; followed by 30 cycles of 94 °C for 30 s, 57 °C for 30 s, and 72 °C for 30 s; and a final extension at 72 °C for 10 min. Three PCR products per sample were pooled to mitigate reaction-level PCR biases. The PCR products were purified using a QIAquick Gel Extraction Kit (Qiagen, Germany), quantified using real-time PCR, and sequenced at Auwigene Company, Beijing. Deep sequencing was performed on the MiSeq platform at Auwigene Company (China). After the reaction, image analysis, base calling, and error estimation were performed using the Illumina Analysis Pipeline Version 2.6.

The fungal ITS region was amplified on an Eppendorf Mastercycler Gradient Thermocycler (Germany) with primers ITS1F (5-GGAAGTAAAAGTCGTAACAAGG-3) and ITS2R (5-ATCCTCCGCTTATTGATATGC-3) [55]. The 5′ ends of both primers were tagged. These ultra-PAGE purified primers were ordered from Majorbio, China. The PCR mixtures were as follows: 4 μL 5× FastPfu Buffer, 1 μL each primer (5 μM), 2 μL dNTP mixture (2.5 mM), 2 μL template DNA, and 10 μL H_2_O. Thermocycling consisted of an initial denaturation at 95 °C for 2 min, followed by 30 cycles of 95 °C for 30 s, 55 °C for 30 s, and 72 °C for 30 s, and then a final extension at 72 °C for 5 min. Three separate reactions were performed to account for potentially heterogeneous amplification from the environmental template for each sample. PCR products were purified using an Axygen Gel Extraction Kit (Qiagen) and quantified using QPCR. An equimolar mix of all three amplicon libraries was used for sequencing at Auwigene Company (China).

### Quality control and analysis of sequence data

The raw sequence data were first screened, and sequences were removed from consideration if they were shorter than 200 bp, had a low-quality score (≤ 20), contained ambiguous bases, or did not exactly match primer sequences and barcode tags. Qualified reads were separated using the sample-specific barcode sequences and trimmed with Illumina Analysis Pipeline Version 2.6. Then, the dataset was analyzed using QIIME. The sequences were clustered into OTUs at a similarity level of 97% to generate rarefaction curves [56] and to calculate the richness and diversity indexes. The Ribosomal Database Project (RDP) classifier tool [57] was used to classify all sequences into different taxonomic groups.

To examine the similarity between different samples, clustering analyses and PCA were used based on the OTU information from each sample using R. The evolution distances between microbial communities from each sample were calculated using a coefficient and represented as an unweighted pair group method with an arithmetic mean (UPGMA) clustering tree describing the dissimilarity (1-similarity) between multiple samples. A Newick-formatted tree file was generated based on this analysis [58]. To compare the memberships and structures of communities in different samples, heat maps were generated with the top 50 OTUs using Mothur.

The fungal raw sequence reads were initially trimmed using Mothur, and sequences that met all three of the following criteria were retained: (1) precise primers and barcodes; (2) quality score > 30; and (3) > 200 bp length. The software package Usearch was then used to further filter sequences that were erroneous or chimeric. The remaining high-quality sequences were queried against the GenBank nonredundant nucleotide database (nt) in NCBI using the local BLASTn. The MEGAN program [59] was used to assign BLAST hits to taxa in NCBI. After removing nonfungal sequence reads, the fungal sequences were clustered into OTUs at a 97% similarity level using UPARSE [60]. Low-abundance OTUs (fewer than 2 reads, including singletons), which might influence richness and diversity estimates, were excluded from subsequent analyses. Rarefaction, richness estimators (ACE and Chao1), and diversity indexes (Shannon and Simpson) of each sample were calculated using Mothur. Weighted and unweighted UniFrac tests were performed using Mothur to determine the statistical significance of structural similarity among communities across sampling locations. Visualization of β diversity information was achieved via ordination plotting with NMDS [28].

### Analysis of the relationships between physicochemical properties and microbial communities

First, according to the overlap between single terminal sequencing sequences (paired end (PE) reads), the MiSeq double terminal sequencing data were spliced using Flash software (version 1.2.3), and then the low-complexity sequences were filtered by quality control with PRINSEQ software (version 0.20.4). The RDP [57] classifier was used to classify the taxa of the processed sequences at the domain, phylum, class, order, family, and genus levels. Data were analyzed using R (v3.6.1). RDA was performed with Canoco 4.5 software to sequence and analyze soil properties and microbial data.

### Biomarker analysis

The taxonomic composition of a microbial community can be influenced by local environmental variables [27]. Thus, the soil microbial communities after different continuous monocropping times should be distinctive, and some bacteria or fungi might be enriched by the different environmental conditions. LEfSe [29] analysis was used to compare data among samples and select biomarkers for each sample. First, ANOVA (analysis of variance) was used to detect species with significant differences in abundance among different samples, and the threshold was set at 0.05. Second, the obtained significantly different species were analyzed by the Wilcoxon rank sum test, and the threshold was set at 0.05. Third, LDA was used to reduce the dimensionality of the data and evaluate the degree of significantly different abundances between species (LDA score), and 3.0 was set as the threshold for the logarithmic LDA score for discriminating features.

## Supplementary information

**Additional file 1: Figure S1.** Top 10 bacterial (A) and fungal (B) phyla identified among the four soil samples. Each stripe denotes the mean of three replicates.

**Additional file 2: Figure S2.** UPGMA tree and principal component analysis of bacterial and fungal communities in soils.

**Additional file 3: Figure S3.** LDA distribution histogram based on LEfSe analysis of classification information for bacterial and fungal communities in soils. Species with differences in LDA scores greater than 3, namely, biomarkers with significant differences, were identified [29]. The length of each bar represents the contribution of species with significant differences in abundance.

**Additional file 4: Table S1.** The identified core OTUs of the bacterial and fungal communities.

## Data Availability

The raw sequencing data have been deposited in the NCBI Sequence Read Archive (SRA) under accession number PRJNA511786.

## Abbreviations

ANOVA: Analysis of variance
AP: Available phosphorus
CK: Control sample
FCC: Fifth-year continuous monocropping
ITS: Internal transcribed spacer
LEfSe: Linear discriminant analysis effect size
NMDS: Nonmetric multidimensional scaling
OM: Organic matter
OTU: Operational taxonomic unit
PCA: Principal components analysis
RDA: Redundancy analysis
RDP: Ribosomal Database Project
SCC: Second-year continuous monocropping
TCC: Third-year continuous monocropping
TN: Total nitrogen
