# Supplementary figures and images for: Diversity patterns of soil microbial communities in the Sophora flavescens rhizosphere in response to continuous monocropping
Source: BMC Microbiol. 2020 Aug 31;20:272. doi: 10.1186/s12866-020-01956-8 (PMC7457492; doi:10.1186/s12866-020-01956-8)

**A**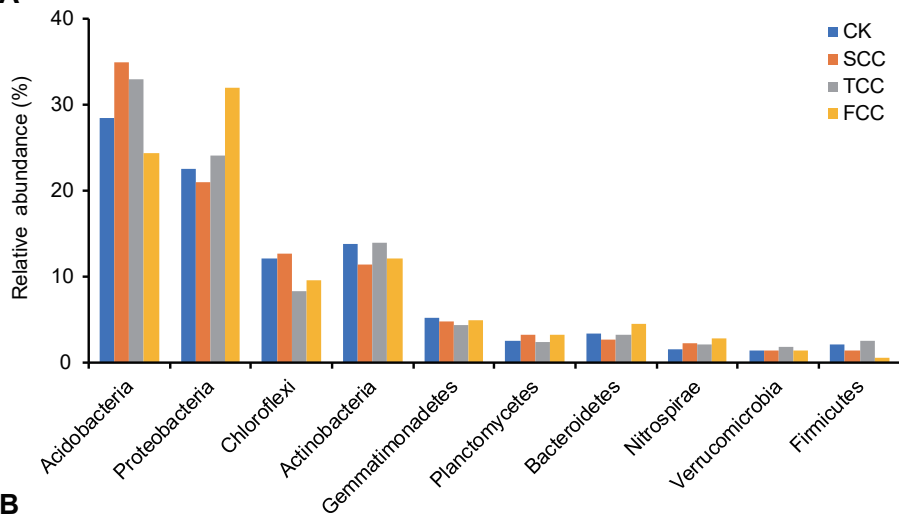**B**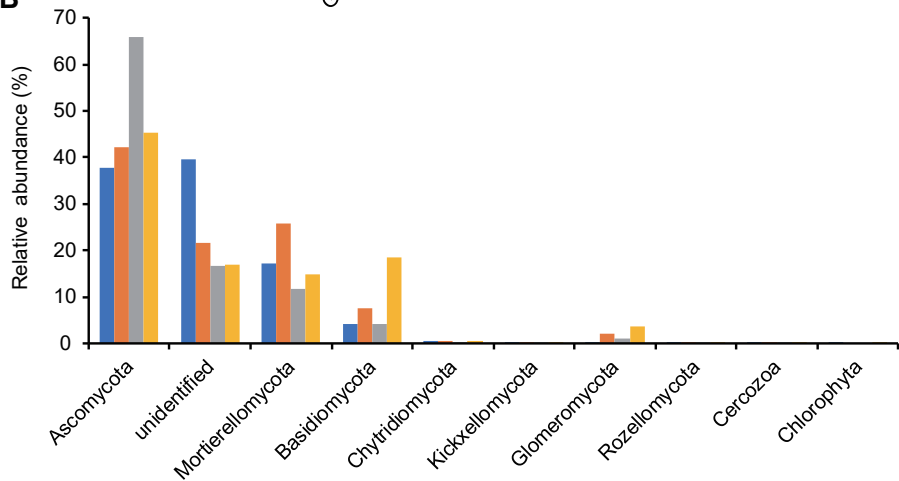

Supplement: Supplementary file 1 — Additional file 1: Figure S1. Top 10 bacterial (A) and fungal (B) phyla identified among the four soil samples. Each stripe denotes the mean of three replicates. [file 12866_2020_1956_MOESM1_ESM.pdf]

**A**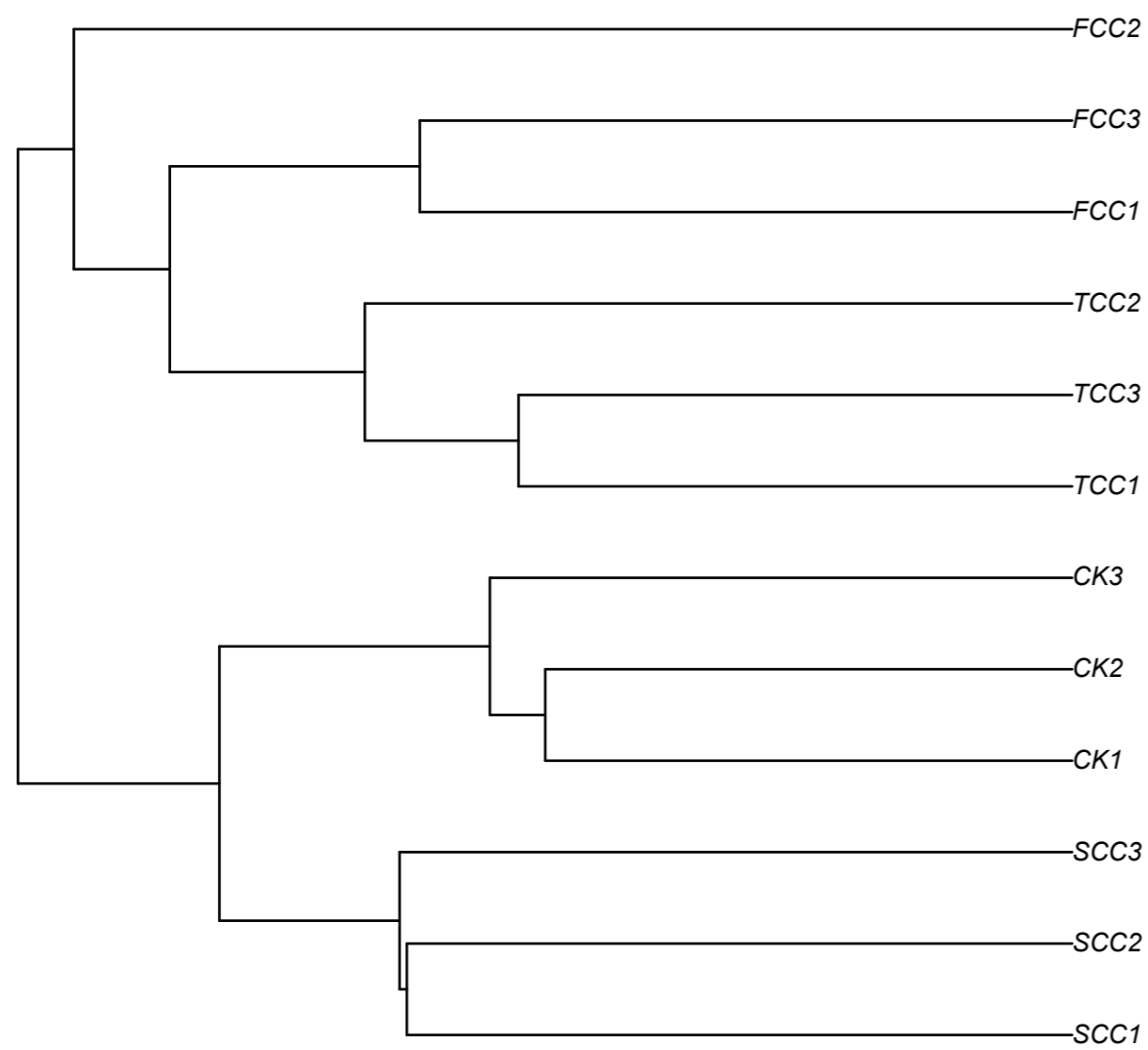**B**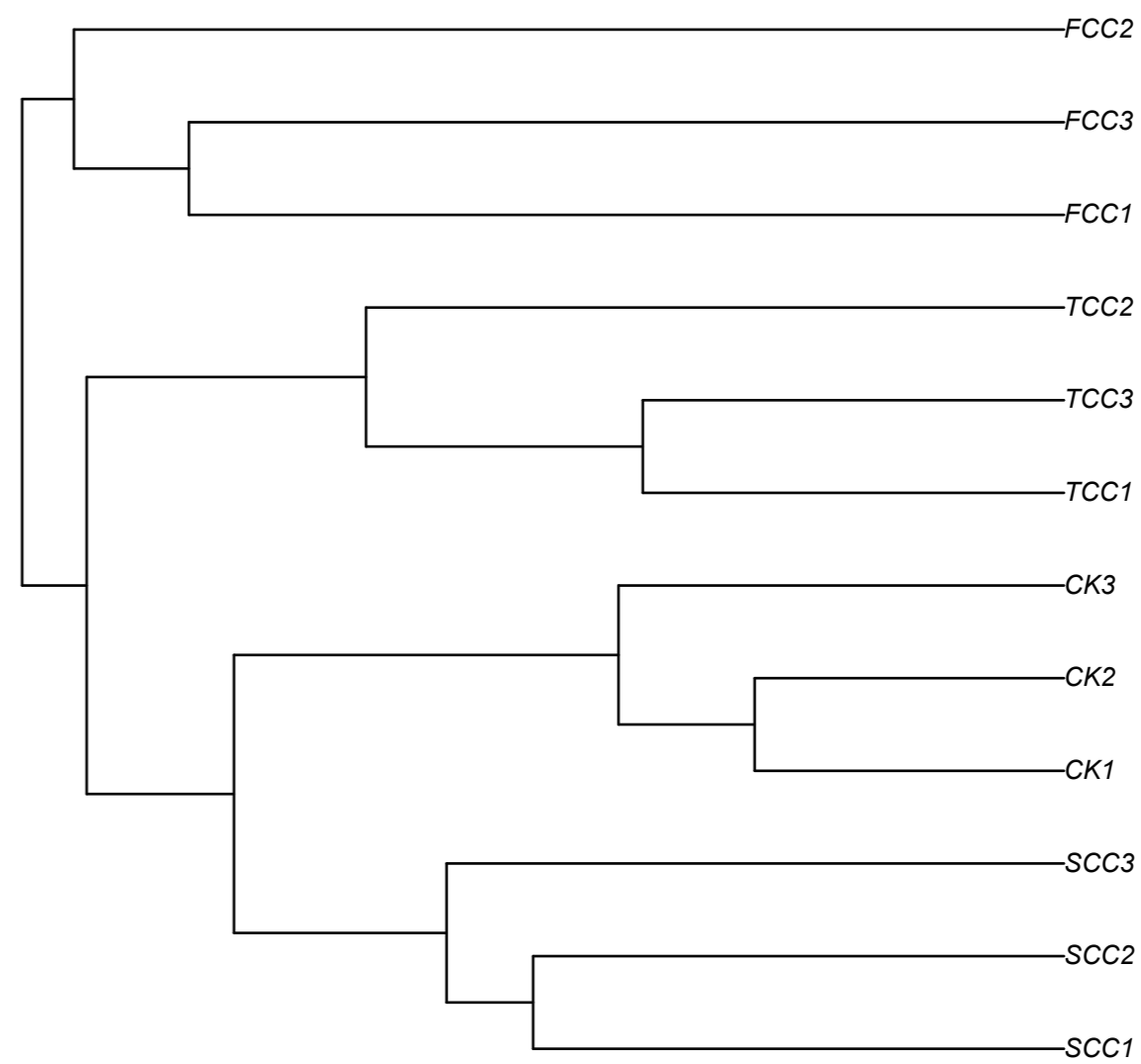**C**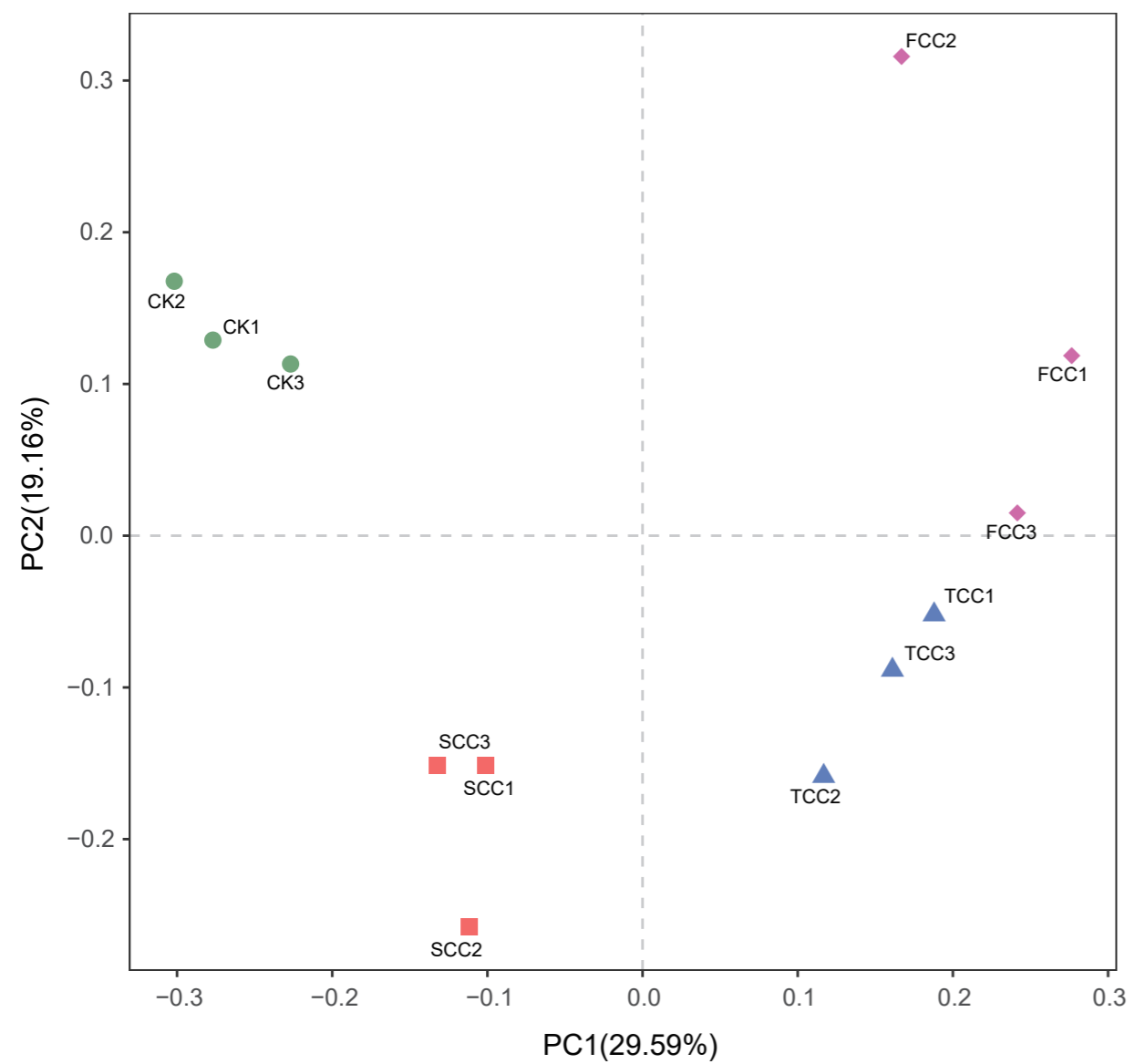**D**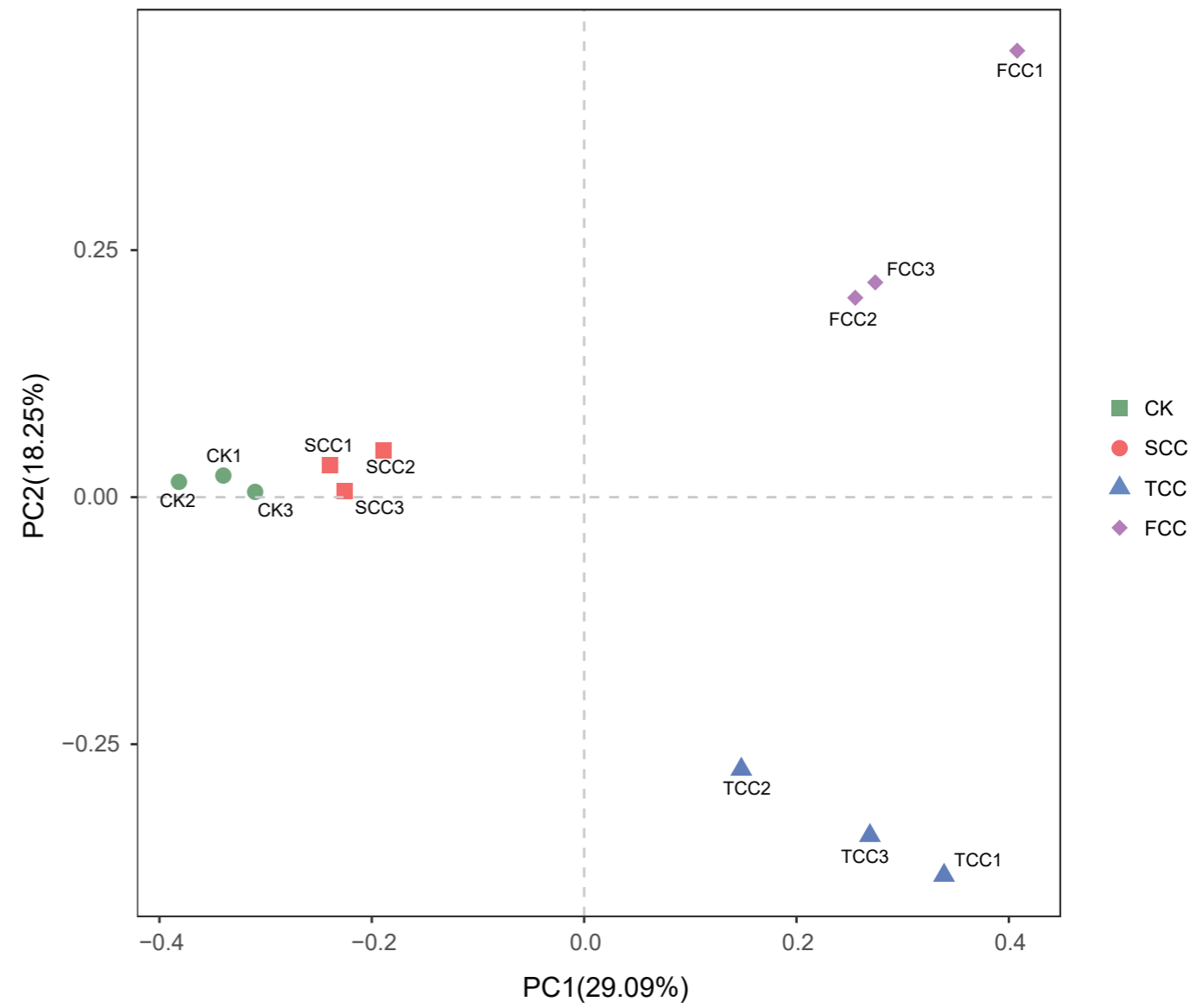

Supplement: Supplementary file 2 — Additional file 2: Figure S2. UPGMA tree and principal component analysis of bacterial and fungal communities in soils. [file 12866_2020_1956_MOESM2_ESM.pdf]

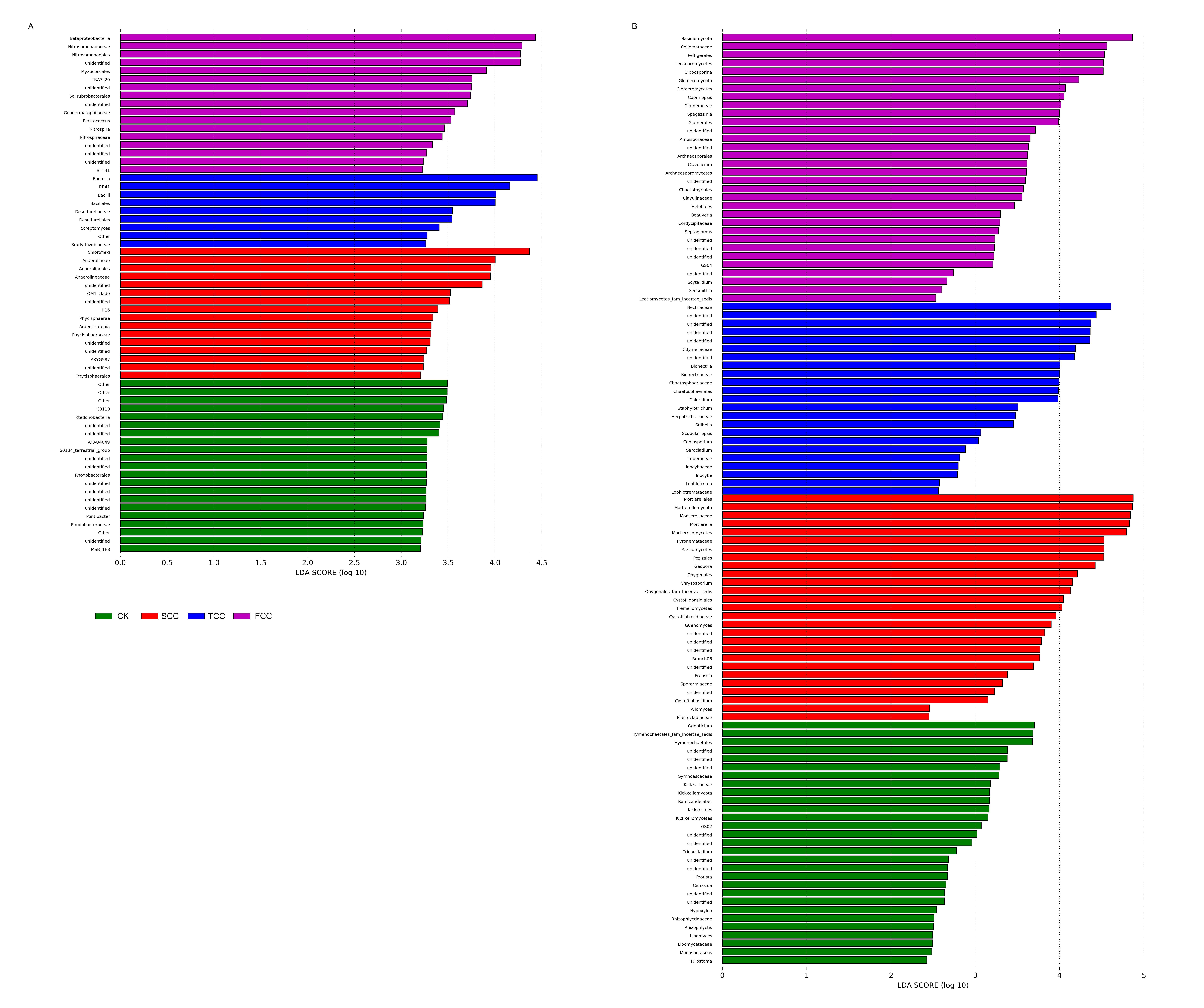

Supplement: Supplementary file 3 — Additional file 3: Figure S3. LDA distribution histogram based on LEfSe analysis of classification information for bacterial and fungal communities in soils. Species with differences in LDA scores greater than 3, namely, biomarkers with significant differences, were identified [29]. The length of each bar represents the contribution of species with significant differences in abundance. [file 12866_2020_1956_MOESM3_ESM.tif]
